# Nonadditive effects of consumption in an intertidal macroinvertebrate community are independent of food availability but driven by complementarity effects

**DOI:** 10.1002/ece3.3841

**Published:** 2018-02-16

**Authors:** Emily M. van Egmond, Peter M. van Bodegom, Jurgen R. van Hal, Richard S. P. van Logtestijn, Matty P. Berg, Rien Aerts

**Affiliations:** ^1^ Department of Ecological Sciences Vrije Universiteit Amsterdam Amsterdam The Netherlands; ^2^ Institute of Environmental Sciences Leiden University Leiden The Netherlands; ^3^ Groningen Institute for Evolutionary Life Sciences, Community and Conservation Ecology Group University of Groningen Groningen The Netherlands

**Keywords:** benthos, community assembly, functional diversity, soft‐sediment beach, trophic interactions

## Abstract

Suboptimal environmental conditions are ubiquitous in nature and commonly drive the outcome of biological interactions in community processes. Despite the importance of biological interactions for community processes, knowledge on how species interactions are affected by a limiting resource, for example, low food availability, remains limited. Here, we tested whether variation in food supply causes nonadditive consumption patterns, using the macroinvertebrate community of intertidal sandy beaches as a model system. We quantified isotopically labeled diatom consumption by three macroinvertebrate species (*Bathyporeia pilosa*,* Haustorius arenarius,* and *Scolelepis squamata*) kept in mesocosms in either monoculture or a three‐species community at a range of diatom densities. Our results show that *B. pilosa* was the most successful competitor in terms of consumption at both high and low diatom density, while *H. arenarius* and especially *S. squamata* consumed less in a community than in their respective monocultures. Nonadditive effects on consumption in this macroinvertebrate community were present and larger than mere additive effects, and similar across diatom densities. The underlying species interactions, however, did change with diatom density. Complementarity effects related to niche‐partitioning were the main driver of the net diversity effect on consumption, with a slightly increasing contribution of selection effects related to competition with decreasing diatom density. For the first time, we showed that nonadditive effects of consumption are independent of food availability in a macroinvertebrate community. This suggests that, in communities with functionally different, and thus complementary, species, nonadditive effects can arise even when food availability is low. Hence, at a range of environmental conditions, species interactions hold important potential to alter ecosystem functioning.

## 1 INTRODUCTION

Although community assembly is in part directly driven by environmental factors, biological interactions are a crucial driver of final community composition (Diamond, 1975; Götzenberger et al., 2012; Valladares, Bastias, Godoy, Granda, & Escudero, 2015). Therefore, the full dynamics and consequences of biological interactions for communities as a whole should be considered, especially in light of environmental change. Changes in environmental conditions affect the interactions between species, and hence final community composition (Griffiths, Warren, & Childs, 2015; Tylianakis, Didham, Bascompte, & Wardle, 2008). When these species and their interactions are subjected to different environmental conditions, a specific set of species is selected for which results in a final community composition which may differ between environmental conditions (Ozinga et al., 2005). As species composition affects community processes (such as consumption) and ultimately ecosystem processes (such as decomposition, primary production, and nutrient cycling), understanding the effects of species interactions on community and ecosystem‐level processes along an environmental gradient is key (Tilman et al., 1997).

Functional differences between species are important determinants of the outcome of their species interactions, resulting in either coexistence or competitive exclusion of species within a community (Chesson, 2000; Valladares et al., 2015). Interactions among organisms may therefore often lead to nonadditive effects of community processes or effects of species composition that cannot simply be explained by expectations based on the species’ monoculture responses (Chapin et al., 2000; Loreau et al., 2001). For example, Cardinale, Palmer, and Collins (2002) showed in a mesocosm experiment that by adding more caddisfly larvae species to a community, facilitative interactions increased, leading to nonadditive effects of resource consumption (i.e., a net diversity effect). Two classes of nonadditive effects can be distinguished and may operate simultaneously: complementarity and selection effects (Huston, 1997; Tilman et al., 1997). Complementarity, selection, and net diversity effects can each be either positive, negative, or zero (Loreau & Hector, 2001). A positive complementarity effect is driven by niche‐partitioning or facilitation (e.g., Spehn et al., 2005; Tilman, Isbell, & Cowles, 2014), while a negative complementarity effect results from physical or chemical interference. The selection effect measures whether differences in species performance in community are nonrandomly related to the performance in monoculture (e.g., Polley, Wilsey, & Derner, 2003). Thus, a positive selection effect occurs when a species with a high monoculture performance is dominant in the community. Complementarity and selection effects together contribute to the net diversity effect. A positive net diversity effect indicates an increased performance of the community, based on the expectation of the monocultures, while a negative effect indicates the opposite. By allocating observed nonadditive effects to these two classes, more insight is gained in which species interactions are involved in, and to what extent they contribute to, a particular community process (Loreau & Hector, 2001). By disentangling whether complementarity or selection effects drive nonadditive consumption in a community, predictions of the effect of changes in community composition on related ecosystem functions can be improved. This makes it a useful tool to assess differences in species interactions within a community under varying environmental conditions.

Laboratory studies of nonadditive effects on community and ecosystem processes have traditionally been conducted under optimal conditions (e.g., Cardinale et al., 2002; Vos, van Ruijven, Berg, Peeters, & Berendse, 2013), with light, nutrients, temperature, and other factors optimized to the needs of the species in the experiment. However, in nature, conditions are rarely optimal and species will have to adapt to survive and maintain in a given community (Berg et al., 2010). Indeed, many studies have shown that environmental stress can significantly impact community and ecosystem processes with a change in species diversity and concomitant changes in ecosystem functioning (e.g., Mulder, Uliassi, & Doak, 2001; Steudel et al., 2012). The impact of different environmental conditions makes it difficult to predict how the outcome of species interactions affects the community as a whole (Loreau, 2000). For example, in a marine benthic food web, the presence of primary and/or secondary consumers led to a change in the trophic cascade, which caused either a positive or negative effect on algal biomass depending on nutrient levels being ambient or enriched (O'Conner & Donohue, 2013). As optimal conditions are uncommon in nature, it is not sufficient to test the effect of nonadditivity on community processes purely under optimal conditions. So far, however, few studies have evaluated how nonadditive effects of consumption change under nonoptimal environmental conditions such as resource availability in a controlled setting.

In this study, we tested how diatom density as a limiting resource impacts community processes using the intertidal macroinvertebrate community of sandy beach ecosystems as a model system. We chose sandy beaches for three main reasons. First, the sandy beach food web is controlled primarily from the bottom‐up and relies heavily on external resource inputs in the form of organic matter (Schlacher & Hartwig, 2013). The intertidal macroinvertebrate community mainly consists of filter and deposit feeders, which depend on microalgae and particulate organic matter (POM) in the water column, which enter the beach at high tide or on benthic microalgae attached to sand grains (McLachlan & Brown, 2006). Secondly, at the sandy beach, food availability is temporally and spatially heterogeneous (Olabarria, Lastra, & Garrido, 2007), for example, due to variable hydrodynamic forces (Menge, 2000; Rosenberg, 1995). This suggests that species will show responses to those different conditions. Finally, the intertidal zone at a sandy beach harbors a macroinvertebrate community with a relatively low complexity, consisting of a limited number of species (Janssen & Mulder, 2005). This allows us to assemble a simple but representative experimental community with results that can be more readily translated to natural field conditions.

The aims of this study were to unravel (i) diatom consumption patterns in a macroinvertebrate community compared to consumption in species’ monocultures upon variation in diatom density supply and, (ii) if observed, differences in diatom consumption at different diatom densities resulted from selection or complementarity effects. To this end, we performed an experiment with isotopically labeled diatoms that were fed to a community, assembled through three macroinvertebrate species commonly found in high abundances within the intertidal community of Dutch beaches (Janssen & Mulder, 2005; Leewis, van Bodegom, Rozema, & Janssen, 2012): *Bathyporeia pilosa* and *Haustorius arenarius* (both amphipods) and *Scolelepis squamata* (a polychaete worm). These species are sand‐dwelling filter and deposit feeders, but differ slightly in feeding strategy making them functionally distinct. *Haustorius arenarius* and *S. squamata* collect any floating particles of organic matter in the water column (Dauer, 1983; Dennell, 1933), while *B. pilosa* mainly scrapes organic material from sand grains (Nicolaisen & Kanneworff, 1969). We focused on three‐species combinations to understand how a simple community of macroinvertebrates would respond to changes in food availability as compared with individual species’ monocultures. We expected that, at high diatom density, interspecific interactions in the macroinvertebrate community would alter consumption compared to the species’ monocultures, because they use the same pool of food (diatoms) and partially overlap in feeding strategy. More specifically, we expected that the individual macroinvertebrate species in monoculture would consume a different amount of diatoms when compared with their consumption in a community. Further, we hypothesized that, if environmental conditions become harsher (when less food in the form of diatoms is available), intertidal macroinvertebrate species with a lower competitive ability would consume less in community compared to that species’ monoculture. As a consequence, selection effects should increase as diatom density decreases. We expected that positive nonadditive effects would occur in our study system, mainly due to niche‐partitioning between the three macroinvertebrate species, promoting coexistence.

## 2 MATERIALS AND METHODS

### 2.1 Experimental design

To investigate the effect of diatom density on consumption by the three macroinvertebrate species both in monoculture and in community, we performed a mesocosm experiment in a fully controlled climate room in which diatom density was manipulated. We used four diatom densities ranging from no diatoms to a high and ad libitum density of diatoms, where in level 0, no diatoms were added, in level 1 and 2, respectively, 10% and 50% of the highest diatom density level were added, and in level 3, 100% of the highest density level was added. Diatoms were offered as a four‐species mixture to ensure a variety of diatoms was available in case the macroinvertebrates would have strong food preferences. Due to practical limitations, we performed our climate room experiment in two parts over time (2‐week in‐between parts), in which all conditions could be kept equal due to the strict environmental controls in the climate room. In both parts, the climate chamber had a 12:12 hours light/dark regime (light intensity: 250 ± 30 μmol m^−2 ^s^−1^, lamp type: Philips Master HPI‐T Plus spectral scheme 50) and a 12:12 hours temperature cycle (20°C during the day and 15°C during the night), ensuring similar environmental conditions. These conditions mimic spring/summer conditions in the Netherlands.

The first experimental part (60 mesocosms in total) contained only monocultures of each macroinvertebrate species to determine diatom consumption at four diatom density levels in the absence of other macroinvertebrate species (Table 1). The second experimental part (35 mesocosms in total) mainly contained communities, consisting of the combination of the three species together, to investigate the effect of diatom density on consumption (Table 1). For this purpose, 20 of these 35 mesocosms contained communities at four diatom density levels. The remaining 15 mesocosms acted as a control. In both experimental parts, there were five replicates for each treatment combination. To confirm there were no differences in macroinvertebrate survival or diatom consumption between both experimental parts (e.g., due to potential differences in environmental conditions or field collection of macroinvertebrates), we included monocultures with the highest diatom density level as a control in the second part. We only duplicated the highest diatom density level to ensure survival and consumption were not affected or limited by a low food availability when including lower diatom densities than ad libitum. There were no significant differences in either survival or diatom consumption per mesocosm for macroinvertebrate monocultures at the highest diatom density level between both parts (Wilcoxon rank‐sum test, *W* = 84.5, *p* = .22; and *W* = 160, *p* = .05, respectively), indicating animal survival and diatom consumption did not depend on the experimental part itself. This allowed us to take both experimental parts together and create one combined dataset for further analysis. Each experimental part lasted 7 days. This time period is sufficient to study short‐term consumption dynamics between these species, as related to the aim of this study.

**Table 1:** Summary of the experimental design indicating the mesocosms included in each experimental part

Experimental part	Monoculture or community	Species	Diatom density (%)	*n*	Total *n*
1	Monoculture	B	0	5	
			10	5	
			50	5	
			100	5	
		H	0	5	
			10	5	
			50	5	
			100	5	
		S	0	5	
			10	5	
			50	5	
			100	5	
					60
2	Community	BHS	0	5	
			10	5	
			50	5	
			100	5	
	Monoculture	B	100	5	
		H	100	5	
		S	100	5	
					35

B, *Bathyporeia pilosa*; H, *Haustorius arenarius*; S, *Scolelepis squamata*.

Mesocosms were constructed of a 30‐cm high PVC tube with a diameter of 11.8 cm, with the lower side closed with a sheet of PVC. A sediment column was constructed in each mesocosm consisting of a layer of 15 cm inert quartz sand (median grain size: 280 μm), followed by a column of 10 cm of artificial seawater (30‰ salt content; Instant Ocean, Aquarium Systems, Inc., Mentor, OH, USA) and, finally, a 5‐cm column of air. During the experiment, all mesocosms were aerated with compressed air via an aeration stone and loosely covered at the top with cling film to limit water evaporation but allowing air exchange (see Figure S1). This mesocosm setup mimics the intertidal beach at high tide, which is when the macroinvertebrate species actively forage on the inundated sand surface and in the water column. In each experimental part, treatments were randomly distributed over the mesocosms and randomly allocated within the climate chamber.

### 2.2 Diatom and macroinvertebrate material

#### 2.2.1 Diatom cultures and stable isotope labeling

Four diatom species covering a range in cell size and life stage (Table 2) were used in the diatom mixture to account for potential feeding preferences of the macroinvertebrates: *Navicula perminuta* (strain CCAP 1050/15) obtained from the Culture Collection of Algae and Protozoa (CCAP, Scottish Marine Institute, United Kingdom), and *Thalassiosira* sp. (strain SCCAP K‐1435), *Amphora* sp. (strain SCCAP K‐1250), and *Skeletonema costatum* (strain SCCAP K‐0669) obtained from the Scandinavian Culture Collection of Algae and Protozoa (SCCAP, University of Copenhagen, Denmark). All four diatom species are commonly found in the North Sea (Ehrenhauss, Witte, Janssen, & Huettel, 2004; Rousseau, Leynaert, Daoud, & Lancelot, 2002; Scholz & Liebezeit, 2012). Diatom cultures were started based upon 20 ml diatom strains within 7 days upon arrival of these strains in separate 500‐ml glass flasks and placed in a climate chamber under optimal conditions. These diatom cultures were tended for approximately 6 months until the start of the experiment to have sufficient diatom culture (2.5–3 L per diatom species in total) available to perform the experiment. Every 4–5 weeks, each diatom culture was subcultured with fresh L1‐medium based on artificial seawater (30‰ salt content; medium protocol obtained from SCCAP) by adding culture to medium in a ratio of 1:10 based on volume.

**Table 2:** Summary of characteristics for the four diatom species used in diatom mixtures, including life stage, cell size (average ± *SD*), and isotopic enrichment for both ^13^C and ^15^N (average ± *SD*)

Species	Life stage	Cell size as length × width (μm)	Experimental part 1		Experimental part 2	
			δ^13^C (‰)	δ^15^N (‰)	δ^13^C (‰)	δ^15^N (‰)
*Navicula perminuta*	Benthic	15.6 ± 4.9 × 2.8 ± 1.6^a^	6,123 ± 19	2,888 ± 6	6,786 ± 71	3,497
*Amphora* sp.	Benthic	76.5 × 17.1^b^	4,769 ± 49	3,087 ± 11	8,000 ± 283	3,689
*Skeletonema costatum*	Planktonic	9.6 × 8.75^c^	9,344 ± 127	3,075	9,483 ± 111	3,353
*Thalassiosira* sp.	Planktonic	21 × 17^d^	6,273 ± 123	2,927 ± 25	6,395 ± 89	3,847 ± 3

Not for each species a duplicate sample could be taken for δ^15^N analysis in each experimental part, therefore lacking a *SD*.

Symbols indicate the following: ^a^Scholz and Liebezeit (2012); ^b^average of four species, Scholz and Liebezeit (2012); ^c^average of two strains, Balzano, Sarno, and Kooistra (2011), ^d^Laws, Pei, and Bienfang (2013).

Diatoms were labeled with the stable isotopes ^13^C and ^15^N to track consumption by the macroinvertebrate species. For ^13^C enrichment, an average of 0.06 g NaH^13^CO_3_ (98% enriched) was added as stock solution per L diatom culture in the last 4 months prior to the experiment in regular intervals. After each ^13^C addition, culture flasks were closed, gently shaken, and left for 4–5 hr to incubate. Flasks were then taken to another room, and the air within the flasks was flushed with compressed air for 15 min to remove surplus ^13^C. For ^15^N diatom enrichment, 0.049 g/100 ml of the N‐source in the regular L1‐medium, NaNO_3_ (7.5 g/100 ml), was replaced by ^15^NH_4_^15^NO_3_ (98% enriched). Final ^13^C and ^15^N diatom enrichment differed between diatom species and both experimental parts (Table 2). For further calculations based on ^13^C and ^15^N diatom enrichment, the average of the four diatom species was taken as we offered diatoms in mixture, with a separate value for each experimental part.

Upon diatom harvest, the culture medium was replaced by fresh artificial seawater to remove nonincorporated stable isotopes. Biomass concentrations were determined by filtering a 50 ml subsample over a 0.45‐μm cellulose nitrate filter, oven‐drying (72 hr at 40°C), and weighing the remaining diatoms. To prepare diatom mixtures, the volume needed from each diatom culture to obtain a 1:1:1:1 dry mass diatom species distribution was taken for each mesocosm according to four diatom density levels. Four diatom density levels were offered to the macroinvertebrates, with the highest diatom density level considered to be ad libitum. In level 0, no diatoms were added (50 ml artificial seawater was added as control) and was considered as a control for isotopic background levels in the macroinvertebrates, level 1 contained 10% of the highest diatom density level (0.0022 g diatoms/50 ml), level 2 contained 50% of the highest diatom density level (0.011 g diatoms/50 ml), and level 3 contained 100% of the highest density level (0.022 g diatoms/50 ml). Level 3 corresponded to two times the average macroinvertebrate biomass of diatoms added per mesocosm (0.010 g animal AFDW). All diatom mixtures were stored cool (5°C) in the dark until the start of the experiment 48 hr later.

#### 2.2.2 Macroinvertebrate collection

Three common macroinvertebrate species from the Dutch intertidal beach (Leewis et al., 2012) were used: *B. pilosa* Lindström, 1855 (Amphipoda: Bathyporeiidae), *H. arenarius* Slabber, 1769 (Amphipoda: Haustoriidae), and *S. squamata* Müller, 1806 (Polychaeta: Spionidae). *Scolelepis squamata* and *H. arenarius* were collected at the beach near Bloemendaal aan Zee (52.42N, 4.55E), the Netherlands, and *B. pilosa* was collected from the Paulinaschor near Terneuzen (51.35N, 3.74E), the Netherlands.

A fresh batch of animals was collected in the field in May 2014, no more than 11 days before the start of each experimental part, by sieving small quantities of hand‐collected sand over a 1‐mm sieve and storing animals in pots containing natural seawater from the collection sites. Collected macroinvertebrates were kept alive in aquaria filled with a 15‐cm layer of inert quartz sand and artificial seawater in the climate chamber. Aquaria were aerated with air stones, and animals were kept under optimal conditions until the start of the experiment. Of the surviving animals that were collected in the field, healthy and active individuals were selected to be randomly divided over the macroinvertebrate treatments. Animals were starved for 24 hr prior to the start of the experiment.

Total macroinvertebrate biomass was kept equal at 0.010 g ash‐free dry weight in each mesocosm, which was based on macroinvertebrate community biomass as found on natural sandy beaches in the Netherlands (personal observations). By dividing by the average dry biomass per individual for each species (adapted from Degraer, Mouton, De Neve, & Vincx, 1999 and Speybroeck, Tomme, Vincx, & Degraer, 2008), the number of individuals per mesocosm was calculated. Densities used in the experiment were within the natural range observed at Dutch sandy beaches (Leewis et al., 2012). To obtain similar biomass in all treatments before the start of the experiment, macroinvertebrate monocultures consisted of nine animals for *B. pilosa* and *H. arenarius* and seven animals for *S. squamata*, whereas the three‐species communities consisted of three *B. pilosa* individuals, three *H. arenarius* individuals, and two *S. squamata* individuals. The measured macroinvertebrate biomass at harvest was used in the calculations on diatom consumption.

### 2.3 Measurements

After 7 days of incubation, all animals were harvested and survival was determined. Animals were retrieved from the mesocosms at harvest and survival was on average 84 ± 19% across all mesocosms, independent of treatment (data not shown). Animals were gently dried with a tissue to remove adherent water and weighed for fresh biomass (to the nearest μg), directly followed by killing the animals by moving them to a vial filled with liquid nitrogen and storing at −80°C until further processing. All individuals per species from one mesocosm were pooled together to have sufficient material for chemical analysis, freeze‐dried for 48 hr, and ground to obtain a homogenized powder. Between 1.0 and 1.5 mg of each sample was weighed in a tin cup for dual (^13^C and ^15^N) stable isotope analysis. Stable isotope enrichment for both diatoms and animals is expressed as a δ value (Fry, 2006). To determine the relative isotope enrichment of the samples, we used Vienna PeeDee Belemnite (VPDB) as the international reference standard for ^13^C and nitrogen in the air for ^15^N (Fry, 2006). The stable isotopes were measured using an elemental analyzer (NC2500; ThermoQuest Italia, Rodana, Italy) coupled with an isotope ratio mass spectrometer (Delta Plus; ThermoQuest Finnigan, Bremen, Germany). For calibration of natural isotope abundance samples, USGS 40 and USGS 41 were used. The reproducibility of the δ^13^ C and δ^15^ N analysis as determined by repeated analysis of an internal standard (Bovine liver, NIST 1577c) was within 0.15 ‰ (*n* = 3). For enriched samples IAEA 305B, IAEA 311(δ^15^ N) and IAEA 309B (δ^13^ C) were used.

To determine initial ^13^C and ^15^N enrichment in the diatoms, two 50 ml subsamples per species were taken and centrifuged at 112 x *g* for 10 min. Each sample was washed two times with artificial seawater (30‰ salt content) to remove nonincorporated stable isotopes present in the medium and dried in the oven (60°C for 72 hr). Diatoms were ground to powder and analyzed for ^13^C and ^15^N with stable isotope analysis. To determine N and C concentrations, powdered diatom samples were washed with demi water to remove salt and then measured by dry combustion with a Flash EA1112 elemental analyzer (Thermo Scientific, Rodana, Italy).

### 2.4 Analysis of diatom consumption

As a measure of diatom consumption, we used the stable isotope enrichment measured in the macroinvertebrates. We determined the isotopic background for both δ^13^C and δ^15^N for each macroinvertebrate species in both monoculture and community at the end of the experiment from the individuals that had been subject to food level 0 (control), where diatoms were absent and thus no isotopic enrichment (i.e., consumption) occurred. The use of species‐specific background δ^13^ C and δ^15^ N accounted for the potential effects of trophic fractionation on stable isotope ratios. In further analysis, we omitted results for 0% diatom density. The difference in δ^13^C and δ^15^N between macroinvertebrates from the other diatom density treatments and the background δ^13^C and δ^15^N provided the assimilated δ^13^C and δ^15^N. In combination with the average freeze‐dry biomass per animal in the mesocosms at harvest, the total assimilation of ^13^C and ^15^N was calculated (in mg per individual). The amount of ^13^C and ^15^N present on average in the diatoms (in mg/mg diatom) was finally used to calculate what diatom mass the macroinvertebrates must have minimally consumed to reach their calculated ^13^C and ^15^N assimilation (in mg diatom/mg animal). This was calculated by dividing the amount of ^13^C and ^15^N in the animals (in mg/animal) by the amount of ^13^C and ^15^N in the diatoms (in mg/diatom) and finally correcting for the mass per individual (in mg/animal) as diatom consumption. Differences in assimilation efficiency among species may have affected these estimates, but these differences are considered to be generally small (Vander Zanden & Rasmussen, 2001). The relatively short experiment does not allow for quantification of stable isotope accumulation within the species’ tissue as full tissue turnover for these species is expected to occur over a longer time period (see e.g., Hentschel, 1998; McLeod, Hyndes, Hurd, & Frew, 2013). Instead, the experiment provided a snapshot of the isotopic dynamics within the animal body between consumption, accumulation, and excretion. Given that this was done for each species after the same incubation period, we expect this to be of only minor influence on the interpretation of the results.

### 2.5 Calculation of diversity effects

The net diversity effect of macroinvertebrate species on consumption was calculated by subtracting the expected consumption in monoculture from the observed consumption in the community at each level of diatom density, for each species separately. The expected diatom consumption for a macroinvertebrate species in community was its consumption in monoculture, adjusted for mass. Under the null hypothesis, the expected and observed diatom consumptions are equal and no selection or complementarity effects occur. To identify the extent to which deviations from this null hypothesis can be attributed to selection or complementarity effects, we used the equation provided by Loreau and Hector (2001). The net diversity effect (Δ*Y*) was the sum of the complementarity effect ($N\bar{\Delta RY}\;\bar{M}$) and the selection effect (*N*_cov_ (∆*RY*,* M*)), with *N* as the number of macroinvertebrate species in the community, ∆*RY* as the difference in relative observed and expected diatom consumption, and *M* as observed diatom consumption in the monoculture. For *M,* we used the average of all mesocosms for each species and food availability combination (*n *=* *5), to have a representative indicator of performance in the mesocosms.

### 2.6 Statistical analysis

We performed a three‐way ANOVA to analyze the overall effect of macroinvertebrate species (three levels), diatom density availability (three levels), and macroinvertebrate community composition (two levels) on diatom consumption. To proceed and to interpret the interactions found, we performed three separate two‐way ANOVA (one for each diatom density level) to test for the effects of macroinvertebrate species and macroinvertebrate community composition on diatom consumption. For each of the two‐way ANOVAs, *p*‐values were corrected for multiple comparisons with the Bonferroni correction, resulting in a *p*_critical_ of .017. Differences in macroinvertebrate composition effect were analyzed with a one‐way ANOVA for net diversity effect, complementarity effect, and selection effect separately and diatom density as a factor. ANOVAs were followed up with Tukey's post hoc tests, which correct for multiple comparisons. There was one outlier in the data; in one, mesocosm consumption was very high for *B. pilosa* in community at 50% diatom density, which could not be rejected on any grounds. However, performing the same analyses for both consumption and diversity effects without this outlier resulted in no changes in accepting or rejecting the tested statistical hypotheses (results not shown). Prior to analysis, all data were tested for homogeneity of variances and a normal distribution. If these assumptions were not met, a square root transformation was performed on the original data (for the three‐way ANOVA, for the two‐way ANOVAs at 10% and 50% food availability, and for net diversity effect and for complementarity effect). *p*‐values were considered to be significant at α = 0.05. All data were analyzed with the statistical program R, version 3.1.2 (R Core Team, 2015).

## 3 RESULTS

### 3.1 Diatom consumption

#### 3.1.1 Overall effects

**Figure 1:**
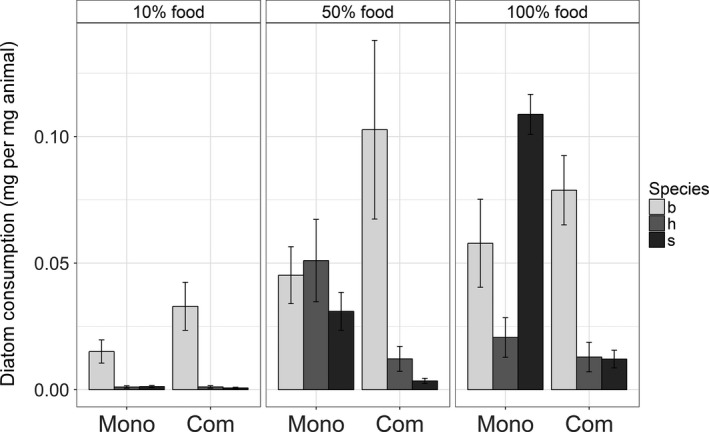
Diatom consumption of three macroinvertebrate species (b, *Bathyporeia pilosa*; h, *Haustorius arenarius*; s, *Scolelepis squamata*) at three different diatom densities (percentage dilution of ad libitum diatom supply) either in monoculture (Mono) or in a three‐species community (Com). Error bars indicate the *SEM*

Diatom consumption by the three macroinvertebrate species over the experimental period varied strongly (maximum consumption was 0.24, 0.10, and 0.14 mg diatom/mg animal for *B. pilosa*,* H. arenarius,* and *S. squamata,* respectively) with diatom density and differed between monocultures and communities (Figure 1). The overall analysis showed that diatom density, macroinvertebrate species, and community composition all had significant effects on diatom consumption. Moreover, all two‐way interactions and the three‐way interaction were significant (see three‐way ANOVA results in Table 3). Below an in‐depth analysis is given by separate comparisons for each level of diatom density.

**Table 3:** Overview of the three‐way ANOVA results on the effect of diatom density, macroinvertebrate species, and community composition on diatom consumption

	*df*	*F*	*p*
Species	2	31.6	<.0001^a^
Diatom density	2	39.3	<.0001^a^
Community composition	1	6.6	.012^a^
Species × diatom density	4	4.4	<.01^a^
Species × community composition	2	18.7	<.0001^a^
Diatom density × community composition	2	3.9	.025^a^
Species × diatom density × community composition	4	3.9	<.01^a^

a Statistically significant *p*‐values at α = 0.05.

#### 3.1.2 High diatom density

Both macroinvertebrate species (two‐way ANOVA, *df* = 2, *F* = 14.1, *p *<* *.001) and community composition (two‐way ANOVA, *df* = 1, *F* = 10.6, *p *<* *.01) had a significant effect on diatom consumption at high diatom density (Figure 1; Table 4). In addition, there was a significant interaction effect between these factors, indicating that macroinvertebrate species consumed differently in the monocultures and in the community (two‐way ANOVA, *df* = 2, *F* = 17.1, *p *<* *.001). In the monocultures, *S. squamata* had the highest diatom consumption with 0.11 ± 0.02 mg diatom/mg animal, followed by *B. pilosa* (0.058 ± 0.039 mg diatom/mg animal; Tukey's post hoc, *p *=* *.02) and *H. arenarius* (0.021 ± 0.018 mg diatom/mg animal; Tukey's post hoc, *p *<* *.001), of which the last two had a similar diatom consumption (Tukey's post hoc, *p *=* *.16). Single species consumption changed when the three macroinvertebrate species were placed together in a community (Figure 1). The average diatom consumption of *S. squamata* dropped sharply by 89% to 0.012 ± 0.008 mg diatom per mg animal as compared to its monoculture (Tukey's post hoc, *p *<* *.001). In contrast, both *H. arenarius* and *B. pilosa* consumed a similar amount of diatoms in both the monoculture and community (Tukey's post hoc, *p *=* *.99 and *p *=* *.72, respectively), with *B. pilosa* having a higher diatom consumption than *H. arenarius* in community (0.079 ± 0.031 against 0.013 ± 0.013 mg diatom per mg animal, respectively; Tukey's post hoc, *p *<* *.01).

**Table 4:** Overview of the two‐way ANOVA results on the effect of macroinvertebrate species and community composition on diatom consumption, for each diatom density level separately

		*df*	*F*	*p*
10% diatom density	Species	2	22.2	<.0001^a^
	Community composition	1	1.0	.324
	Species × community composition	2	1.5	.237
50% diatom density	Species	2	10.3	<.001^a^
	Community composition	1	2.6	.117
	Species × community composition	2	7.9	<.01^a^
100% diatom density	Species	2	14.1	<.0001^a^
	Community composition	1	10.6	<.01^a^
	Species × community composition	2	17.1	<.0001^a^

For each of the two‐way ANOVAs, *p*‐values are corrected with the Bonferroni correction at α = 0.05, resulting in a *p*_critical_ of .017.

a Statistically significant *p*‐values at *p*_critical_ = .017.

#### 3.1.3 Low diatom densities

Diatom consumption by the three macroinvertebrate species was altered considerably at low diatom densities compared to high diatom densities (Figure 1, Table 4). At 50% diatom density, macroinvertebrate species differed significantly in diatom consumption (two‐way ANOVA, *df *= 2, *F* = 10.3, *p *<* *.001) and there was a significant interaction effect between species and community composition (two‐way ANOVA, *df *= 2, *F* = 7.9, *p *<* *.01). Interestingly, each macroinvertebrate species consumed a similar amount of diatoms in the community and its respective monoculture (Tukey's post hoc, *B. pilosa*:* p *=* *.23; *H. arenarius*:* p *=* *.14, and *S. squamata*:* p *=* *.15), even though *H. arenarius* and *S. squamata* appeared to have consumed less in the community (see Figure 1). This nonsignificant difference is possibly due to the large variation observed in diatom consumption for *B. pilosa* and *H. arenarius*, both in monoculture and community. In the community, *B. pilosa* had a higher diatom consumption compared to *H. arenarius* (Tukey's post hoc, *p *<* *.01) and *S. squamata* (Tukey's post hoc, *p *<* *.001). Regardless of community composition, *B. pilosa* was the only species that significantly affected diatom consumption at 10% diatom density, while *H. arenarius* and *S. squamata* showed little response in terms of consumption (ANOVA, *df *= 2, *F* = 22.2, *p *<* *.001; Figure 1, Table 4). The amount of diatoms consumed by *B. pilosa* was not different between its monocultures and in community at 10% diatom density (Tukey's post hoc, *p *=* *.37).

### 3.2 Net diversity effect of macroinvertebrate diversity and its components

**Figure 2:**
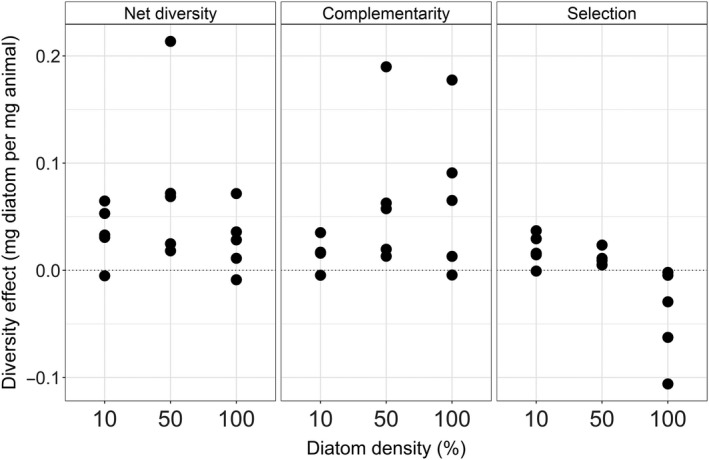
Net diversity effect, complementarity effect, and selection effect as function of diatom density (percentage dilution of ad libitum diatom supply), expressed as the amount of diatoms consumed (mg per mg animal). Each dot represents one mesocosm containing a three‐species community

The net diversity effect on consumption was positive and similar across diatom densities (one‐way ANOVA, *df *= 2, *F *= 1.5, *p *=* *.27) and was mainly driven by complementarity effects. This is clearly shown by the more positive complementarity effect compared to the selection effect, which was closer to zero or negative (Figure 2). Differences in diatom consumption between what we expected from the species’ monocultures and observed in the community were thus negligible between high and low diatom densities. The complementarity effect did not significantly change with diatom density (one‐way ANOVA, *df* = 2, *F* = 1.4, *p *=* *.29). However, the selection effect did change from predominantly negative at 100% diatom density to mildly positive when fewer diatoms were available (one‐way ANOVA, *df *= 2, *F* = 7.2, *p *<* *.01). Thus, selection effects contributed more to the overall net diversity effect with decreasing diatom density. For the net diversity effect, the selection effect finally was canceled out by a slightly higher complementarity effect at high diatom density (Figure 2).

## 4 DISCUSSION

In this study, we document a strong influence of diatom density on diatom consumption by intertidal macroinvertebrate species, both for single species and in species mixtures. These macroinvertebrate species showed a different consumption pattern when diatom densities decreased. Diatom consumption was obviously lower when food was scarce, but importantly, the nature of macroinvertebrate species interactions changed as well. Even though the three macroinvertebrate species responded differently under varying diatom densities, the net diversity effect on consumption was positive and similar across all diatom densities. A positive net diversity effect indicates an increased performance of the community based on the expectation of the monocultures. Complementarity in consumption was the main driver of the net diversity effect, with an increasing contribution of selection effects with decreasing diatom densities. Hence, the nonadditive effects on consumption observed in this intertidal macroinvertebrate community were independent from diatom density and concomitant changes in macroinvertebrate species interactions. This suggests that, in communities with functionally different, and thus complementing species in terms of feeding strategy, nonadditive effects can arise even when food availability is low.

### 4.1 High diatom densities: *B. pilosa* is the most successful consumer

As expected, diatom consumption at high diatom density was altered in community as compared with the species’ monocultures, with *B. pilosa* having highest consumption in the community, while *S. squamata* consumed most in monoculture. One explanation is that *B. pilosa* is “released” from intraspecific competition in the community as it is surrounded by fewer conspecifics, potentially making it a more successful competitor with other species. *Bathyporeia pilosa* has a preference for benthic microalgae (Maria, De Troch, Vanaverbeke, Esteves, & Vanreusel, 2011) and feeds by scraping the organic material from sand grains (Nicolaisen & Kanneworff, 1969). In contrast, *H. arenarius* and *S. squamata* both have a less distinguished preference for food particles (Dauer, 1983; Dennell, 1933). Assuming that *B. pilosa* as a specialist is more efficient in consuming its preferred food source (Büchi & Vuilleumier, 2014), for example, by lower handling times or higher ingestion rates, it will reduce food availability for the other species in the community.

In general, the specialist *B. pilosa* is most successful in community, but not the most successful species in monoculture at high food conditions, which is the generalist *S. squamata*. These findings are in accordance with theory (Büchi & Vuilleumier, 2014), stating that specialists outcompete generalists in their optimal habitat (here: high benthic diatom density at 100% diatom density) and that heterogeneity of food together with the absence of strong competing species (here: in monoculture) favors generalists. A shift from a single species assemblage to a community thus coincides with a change in total consumption by the individual macroinvertebrate species when food availability is high and heterogeneous.

### 4.2 Low diatom densities: *B. pilosa* remains the most successful consumer

The species with the lowest competitive ability in this experiment, *S. squamata*, consumed slightly less in community when diatom density decreased. Although the effect is relatively small, it does support our hypothesis. At lower diatom densities, we found that *B. pilosa* remained the most successful species in terms of consumption, with a higher consumption than *S. squamata* and *H. arenarius* in community. At 50% diatom density, the three species consumed similar amounts of diatoms in monoculture. Both *H. arenarius* and *S. squamata* consumed less in community compared to their respective monocultures, but the difference in consumption is slightly smaller for *H. arenarius*. The main feeding strategy of *H. arenarius* is to move the water with its maxillae and filter very small organic particles from this (Dennell, 1933). In contrast, *S. squamata* feeds primarily on larger particles (Dauer, 1983). *Haustorius arenarius* therefore appears better capable of using smaller food particles than *S. squamata*, potentially explaining its slightly higher consumption than *S. squamata* in community. At 10% diatom density, only *B. pilosa* was able to feed on diatoms. *Haustorius arenarius* and *S. squamata* were unable to consume at this low diatom density, even in the absence of other macroinvertebrate species. In monoculture, these two macroinvertebrate species possibly had difficulty discovering the food as there were few food particles available, resulting in no consumption. *Bathyporeia pilosa* consumed a similar amount of diatoms in community as in monoculture, providing another piece of evidence that interspecific competition did not affect its consumption when food was scarce. The patterns found in diatom consumption coincide with macroinvertebrate occurrences within the tidal regimes of sandy beaches. *Bathyporeia pilosa* resides mostly in the higher part of the intertidal zone where food supply is low (Fish & Preece, 1970; Van Tomme, Van Colen, Degraer, & Vincx, 2012), while *H. arenarius* and *S. squamata* are generally found in the midtidal part of the intertidal zone (Leewis et al., 2012) where food availability may locally be enhanced by seawater containing microalgae (McLachlan & Brown, 2006).

Thus, in this experiment, the specialist, *B. pilosa*, remained the better competitor at low diatom densities. This is slightly unexpected, because a specialist is predicted to show lower consumption when preferred food is encountered on a less predictable basis (Büchi & Vuilleumier, 2014), although specialists are also expected to be more efficient consumers of the (low amounts of) food available. As a consequence, consumption patterns across the species did not change when food availability is low.

### 4.3 A consistent nonadditive effect, but shifts between selection and complementarity effects

#### 4.3.1 Complementarity effects

In accordance with our expectation, we found a large and dominant‐positive complementarity effect across diatom densities, indicating that functionally distinct species can increase total consumption. Functionally different species may be better able to coexist due to stabilizing niche differences, such as niche‐partitioning, when these effects are greater than relative fitness differences (HilleRisLambers, Adler, Harpole, Levine, & Mayfield, 2012; Tilman et al., 1997; Valladares et al., 2015), making niche‐partitioning a probable mechanism of enhancing total diatom consumption in this study system. The different feeding strategies of these co‐occurring macroinvertebrate species in the intertidal beach make them functionally different in terms of consumption. This difference accounted for part of the variation in consumption observed in this study. Facilitation is another aspect of complementarity, but, in intertidal communities, the main facilitation mechanism appears to be the amelioration of physical stress (e.g., temporal drought and reworking of soft sediments) (Bulleri, 2009). This is not directly linked to food availability and biological interactions and is in general unlikely to occur in this mesocosm setup with identical physical environments. Therefore, niche‐partitioning appears to be the most probable mechanism explaining observed positive complementarity effects in our experiment (Finke & Snyder, 2008). Although previous studies reported an increase in the positive complementarity effect for ecosystem functioning with an increase in resource availability (Boyer, Kertesz, & Bruno, 2009; Fridley, 2002), we did not find this pattern. Given the short time period of our study, we used consumption as the response variable, which is a more constrained response variable as individuals reach satisfaction on a shorter timescale than, for example, for a variable as biomass production. Indeed, the highest food level in our experiment was considered to be ad libitum; thus, an excessive food supply does not necessarily lead to increased consumption. Consequently, positive complementarity effects do not necessarily increase with an increase in resource availability. If the enhanced total consumption in community is however maintained over a longer time period, this could lead to a higher intertidal macroinvertebrate biomass and promote the flow of nutrients in the intertidal sandy beach ecosystem.

#### 4.3.2 Selection effects

We hypothesized that selection effects would increase with declining diatom densities, which we indeed observed. Selection effects were mainly negative at high diatom density and mildly positive at low diatom density, which is in accordance with theoretical expectations (Loreau, 2000). When less food is available, there appears to be a slightly higher impact of competition between macroinvertebrate species on consumption when they have to use the same food source and when their niches are not fully partitioned. At low diatom densities, relative fitness differences, for example due to different resource requirements, become more important and influence competitive interactions (HilleRisLambers et al., 2012). However, it seems unlikely that a cascading effect of potential differences in relative fitness on total consumption occurred, as consumption was similar between monocultures and the community.

#### 4.3.3 Total net biodiversity effect

The net diversity effect on consumption was positive and similar across diatom densities and was mainly driven by complementarity effects. Although we found that, at 10% and 50% diatom density, the contribution of the less successful species *H. arenarius* and *S. squamata* to the total consumption in community was limited, consumption was always greater in the community as compared with the expectation based on the species’ monocultures. This shows that the range of functions across macroinvertebrate species was large enough to observe nonadditive effects for consumption and therefore increase resource use efficiency. *Bathyporeia pilosa* contributed most to the total net biodiversity effect of consumption and was also the species with the lowest average biomass (1.38 ± 0.23 mg dry biomass against 5.69 ± 0.86 mg and 7.58 ± 2.83 mg for *H. arenarius* and *S. squamata,* respectively). However, it is expected that species with a high biomass exert the largest effect on consumer performance and related community processes (Reiss, Bailey, Perkins, Pluchinotta, & Woodward, 2011), and this discrepancy may be due to a higher mass‐specific metabolic rate of small‐bodied organisms such as *B. pilosa*.

### 4.4 Implications for community ecology

The differences in consumption between macroinvertebrate species within a community, as found here, could eventually lead to changes in community composition. In turn, this altered community may influence food availability via consumption. This touches upon the central question of whether species diversity is a consequence of (response) or a cause of (effect) resource availability, and these effects have been shown to be able to occur simultaneously (Cardinale, Weis, Forbes, Tilmon, & Ives, 2006). However, we only tested for the former aspect. Following community dynamics over time and tracking both resource density and consumer composition may help to clarify how these are linked (and intertwined). Finally, when translating our findings to field conditions, the impacts of differences in environmental conditions need to be accounted for. Diatom density is a direct result of marine primary production which is an important ecosystem function. Diatom production can be enhanced, for example, by an increase in anthropogenic nutrient input which is currently widespread in marine systems (Allgeier, Rosemond, & Layman, 2011) or reduced by overconsumption (e.g., Schlacher & Hartwig, 2013). Wave and current strength subsequently may affect diatom supply to the beach, creating resource heterogeneity that influences the relation between species composition and community and ecosystem processes (Dyson et. al., 2007), while hydrodynamics may as well directly affect consumption patterns. Despite these limitations to our current setup, we predict that higher macroinvertebrate functional dissimilarity results in a higher total consumption by the community. In a bottom‐up controlled ecosystem, high consumer dissimilarity thus aids in making nutrients available from primary production to higher trophic levels.

## 5 CONCLUSIONS

In the context of community responses to differences in environment, we showed that variation in food availability consistently leads to positive nonadditive effects of consumption by a macroinvertebrate community. This suggests that, in communities with functionally different, and thus complementing, species, nonadditive effects can arise even when food availability is low. This finding is especially relevant in ecosystems (such as coastal or tundra ecosystems) where food supply is limited or variable, either because of temporal or spatial variability, and the ecosystem is primarily bottom‐up controlled.

## CONFLICT OF INTEREST

We have no conflict of interest to declare.

## AUTHOR CONTRIBUTIONS

EME, PMB, MPB, and RA conceived and designed the experiment. EME, JRH, and RSPL performed the experiment. EME, PMB, MPB, and RA analyzed the data. EME wrote the manuscript with the other authors providing editorial advice.

## DATA ACCESSIBILITY

Data of this paper have been deposited in the Dryad repository: https://doi.org/10.5061/dryad.t39g3 (van Egmond et al. 2018).
