# Supplementary material for: Nonadditive effects of consumption in an intertidal macroinvertebrate community are independent of food availability but driven by complementarity effects
Source: Ecol Evol. 2018 Feb 16;8(6):3086–97. doi: 10.1002/ece3.3841 (PMC5869218; doi:10.1002/ece3.3841)
Supplement: Supplementary file 1 [file ECE3-8-3086-s001.docx]

**Supplementary material**


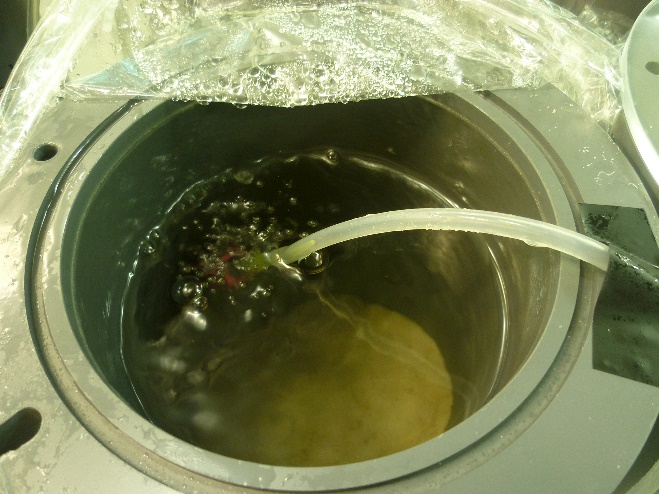


A


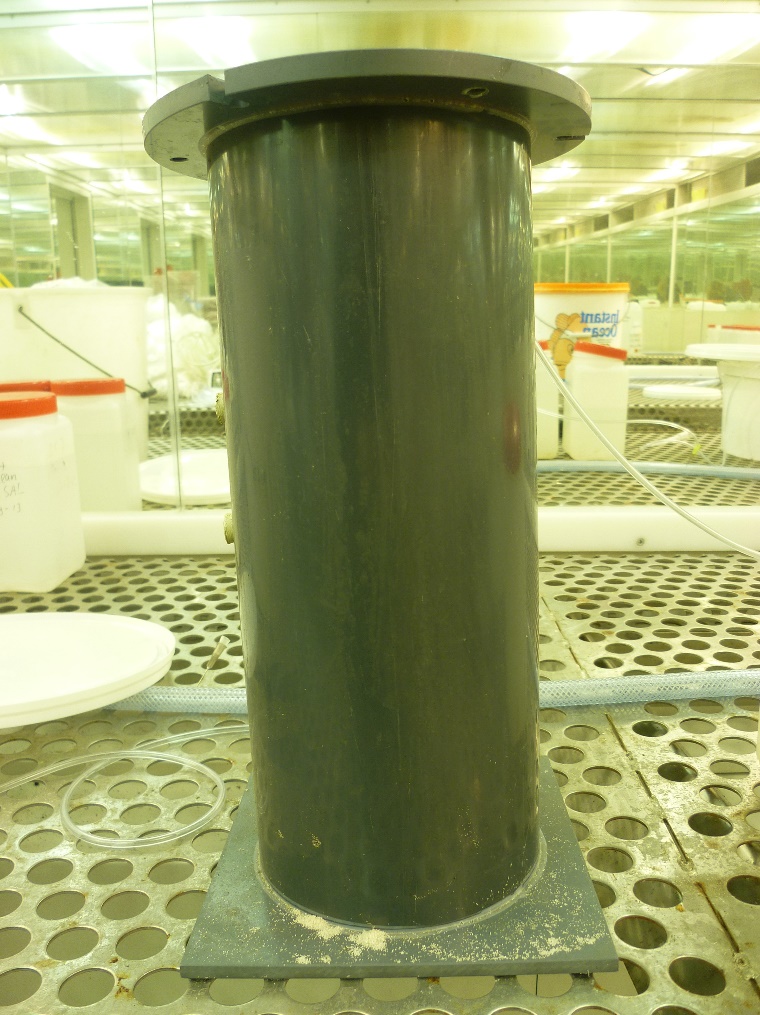


B


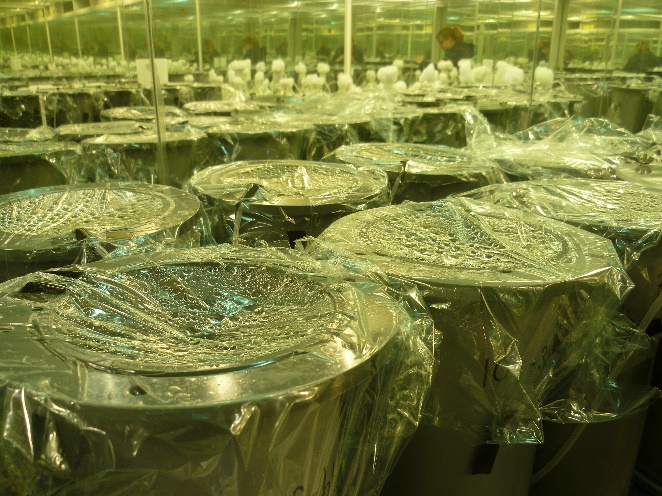


C

**Figure S1.** Three pictures showing the mesocosms used in the experiment: A) a helicopter-view of an open mesocosm (plastic foil removed) filled with sand and water, with aeration of the water column in action, B) side view of a mesocosm and C) an overview of the mesocosms as placed in the climate room during the experiment.
